# Systematic identification and stratification of help-seeking school-aged youth with mental health problems: a novel approach to stage-based stepped-care

**DOI:** 10.1007/s00787-021-01718-5

**Published:** 2021-01-18

**Authors:** Rasmus Trap Wolf, Louise Berg Puggaard, Mette Maria Agner Pedersen, Anne Katrine Pagsberg, Wendy K. Silverman, Christoph U. Correll, Kerstin Jessica Plessen, Simon-Peter Neumer, Dorte Gyrd-Hansen, Mikael Thastum, Niels Bilenberg, Per Hove Thomsen, Pia Jeppesen

**Affiliations:** 1 Child and Adolescent Mental Health Centre, Mental Health Services – Capital Region of Denmark, Copenhagen, Denmark; 2 Department of Clinical Medicine, Faculty of Health and Medical Sciences, University of Copenhagen, Copenhagen, Denmark; 3 Danish Centre for Health Economics, Department of Public Health, University of Southern Denmark, Odense, Denmark; 4 Division of Child and Adolescent Psychiatry, Department of Psychiatry, Lausanne University Hospital CHUV, Lausanne, Switzerland; 5 Department for Child and Adolescent Psychiatry, Mental Health Services in the Region of Southern Denmark, Copenhagen, Denmark; 6 University of Southern Denmark, Odense, Denmark; 7 Research Center At Department for Child- and Adolescent Psychiatry, Aarhus University Hospital, Skejby, Denmark; 8 Institute of Clinical Medicine, Faculty of Health, Aarhus University, Aarhus, Denmark; 9 Centre for the Psychological Treatment of Children and Adolescents, Department of Psychology and Behavioural Sciences, Aarhus BSS, Aarhus University, Aarhus, Denmark; 10 Centre for Child and Adolescent Mental Health, Oslo, Norway; 11 Centre for Child and Youth Mental Health and Child Welfare, The Arctic University of Norway, North Norway (RKBU North), Tromsø, Norway; 12 Anxiety and Mood Disorders Program, Yale Child Study Center, School of Medicine, Yale University, New Haven, CT USA; 13 Department of Psychiatry and Molecular Medicine, Donald and Barbara Zucker School of Medicine At Hofstra/Northwell, Hempstead, NY USA; 14 Department of Psychiatry, The Zucker Hillside Hospital, Glen Oaks, NY USA; 15 Center for Psychiatric Neuroscience, Feinstein Institute for Medical Research, Manhasset, NY USA; 16 Department of Child and Adolescent Psychiatry, Charité Universitätsmedizin, Berlin, Germany

**Keywords:** Mental health services, Mental health problems, Stage-based stepped-care, Visitation, Youth, Children and adolescents

## Abstract

We investigated whether a novel visitation model for school-aged youth with mental health problems based on a stage-based stepped-care approach facilitated a systematic identification and stratification process without problems with equity in access. The visitation model was developed within the context of evaluating a new transdiagnostic early treatment for youth with anxiety, depressive symptoms, and/or behavioural problems. The model aimed to identify youth with mental health problems requiring an intervention, and to stratify the youth into three groups with increasing severity of problems. This was accomplished using a two-phase stratification process involving a web-based assessment and a semi-structured psychopathological interview of the youth and parents. To assess problems with inequity in access, individual-level socioeconomic data were obtained from national registers with data on both the youth participating in the visitation and the background population. Altogether, 573 youth and their parents took part in the visitation process. Seventy-five (13%) youth had mental health problems below the intervention threshold, 396 (69%) were deemed eligible for the early treatment, and 52 (9%) had symptoms of severe mental health problems. Fifty (9%) youth were excluded for other reasons. Eighty percent of the 396 youth eligible for early treatment fulfilled criteria of a mental disorder. The severity of mental health problems highlights the urgent need for a systematic approach. Potential problems in reaching youth of less resourceful parents, and older youth were identified. These findings can help ensure that actions are taken to avoid equity problems in future mental health care implementations.

**Supplementary Information:**

The online version contains supplementary material available at 10.1007/s00787-021-01718-5.

## Introduction

A large proportion of children and adolescents worldwide suffer from mental disorders [1]. A recent nationwide study reported a cumulative treatment incidence of 15% for any mental disorder before the age of 18 years in Denmark [2]. Child and Adolescent Mental Health Services (CAMHS) in Denmark and other countries worldwide are faced with a huge demand for diagnostic assessment procedures and effective treatments [3–5]. The urgent need for safe and effective interventions targeting child and adolescent (youth) emotional and behavioural problems is accentuated by the negative individual and societal consequences associated with early mental health problems. These include putting the youth on a trajectory for chronic mental disorders with the consequence of impaired daily functioning, educational and social difficulties, and absence from the job market in adulthood [6]. Such negative consequences are also present for subthreshold conditions, which are linked to many of the same negative long-term outcomes [6], including a higher risk of suicide [7]. With most mental disorders presenting first in childhood and adolescence [8], prevention and timely delivery of care are solutions to lowering the rates of adult mental health disorders and to meet the needs of the youth suffering from disorders and subthreshold conditions.

Several national health bodies thus recommend a stepped-care approach in child and adolescent mental health [9–11]. The stepped-care approach aims to offer interventions in increasing intensity levels by offering low-intensity interventions as the first step, and subsequently interventions with higher intensity. The goal is to minimize intervention costs and time burden for youth and their parents are kept as minimal as possible, while still offering evidence-based interventions [12]. Stepped-care approaches have been criticized for potentially delaying appropriate treatment by having individuals go initially through insufficient low-intensity interventions [9]. The approach has also been criticized for not fitting ‘real-world’ settings in that youth do not usually present with well-defined single disorders or conditions that are easily recognized and matched to a particular step of care [9, 13]. Thus, it is often unclear when and to whom specific interventions should be given in a stepped-care approach [14], as the diagnostic thresholds can be too arbitrary to define treatment and treatment intensity indication [9]. As an answer to this criticism, a stage-based stepped-care approach has been discussed and developed [9, 15]. Staging, as known from general medicine, is based on placing the problem or condition on a continuum of stages that are defined by the available interventions, clarifying the match of problems and interventions [16]. Furthermore, the stage-based stepped-care approach allows for the complexity of children’s and adolescents’ mental health problems by not necessarily focusing on specific diagnoses [15].

Despite the appeal of the stage-based stepped-care approach, it is still not widely implemented for school-aged children and adolescents. For such an approach to work in clinical practice, there is a need for a coherent process from early identification and systematic stratification to interventions of all intensities. In many countries, mental health care is divided so that prevention and general counselling is located outside the health care system in schools and community services under local governments, while diagnostic assessment and treatments are carried out in hospital or outpatient clinics [17]. With increasing rates of families seeking help for youth mental health problems and disorders, this dichotomy creates a gap in care for youth who need more help than community counselling, but less than specialized CAMHS treatment. For example, 27% (*N* = 1566) of all referrals to CAMHS in the Capital Region of Denmark were rejected in 2018 (personal communication). This may cause a vicious cycle in which youth with milder conditions must either wait for their conditions to deteriorate before they can get appropriate care, or they never receive care despite the risk of negative long-term consequences [6, 7]. When applied to internal medicine, this current approach corresponds to not treating a person with high blood pressure before a cardiac infarct has manifested.

Evidence-based programs for anxiety, milder depression, and behavioural problems and disorders in children and adolescents exist [18–21], but are not systematically offered. This lack of implementation may be partly due to the high costs and logistic difficulties in training and implementation of several diagnosis specific programs. These are problems that can potentially be addressed by the development of effective transdiagnostic programs [22]. The Mind My Mind (MMM) intervention is a transdiagnostic and modular cognitive and behavioural intervention for school-aged youth who have clinically significant levels of emotional and/or behavioural problems that impact their daily lives and threaten to disrupt their development, but who do not qualify for treatment in CAMHS. The intervention aims at providing early treatment of anxiety, depressive symptoms and/or behavioural problems. The MMM trial is a pragmatic, multi-site, randomized, parallel-group, controlled trial of the MMM intervention versus management as usual (MAU).

For the purpose of identifying youth in need of an intervention for emotional and/or behavioural problems and to prepare for dissemination of evidence-based treatment, a standardized visitation model was developed and implemented in four municipalities across Denmark. The model aimed to stratify the help-seeking youth into three groups with increasing severity of mental health problems based on a stage-based stepped-care principle. Hence, the groups with increasing severity of mental health problems were defined based on the possible actions: (Stage 1) self-help and general counselling in the municipalities, (Stage 2) early treatment for mental health problems, in this cases, in the form of participation in the MMM trial, and (Stage 3) referral to CAMHS or other health care professionals for more comprehensive assessment and treatment.

In this study, we had two primary aims: (1) To describe the help-seeking population and the visitation model based on psychopathological questionnaires, and (2) To analyse the representativeness of the population in the visitation compared to the background population in the four municipalities using unique individual-level data on socio economic factors from national registers. Hence, we investigated whether the visitation model, when operating in a naturalistic setting, facilitated a systematic identification and stratification process with equal access to care. We further examined the feasibility of the visitation process by analysing the duration and mean changes in psychopathology for the youth evaluated a second time in relation to the baseline of the MMM trial.

## Method

### Setting

The MMM trial was conducted in four Danish municipalities located in three of the five regions in Denmark. The procedures that led to inclusion in the trial took place from 7th September 2017 till 18th December 2018. The details of the intervention are described in the online study record (NCT03535805).

The trial was implemented in the context of Educational and Psychological Counselling Services (PPR) in the municipalities. PPR psychologists conducted the visitation and intervention whilst supervised by a senior consultant in child and adolescent psychiatry (author PJ).

### Assessment of psychopathology

The Strengths and Difficulties Questionnaire (SDQ) is a widely used and well-validated tool to identify and assess children with mental health problems in both clinical samples and in general population-based samples [23–25]. SDQ is available in validated versions for parents and for youth ≥ 11 years old. The questionnaire contains 25 items, which cover five subscales relating to the children’s Emotional problems, Peer problems, Behavioural problems, Hyperactivity and Pro-social behaviour. Responses to the first four subscales are summarized to calculate a total difficulties score. Each subscale score ranges from 0 to 10, so that the Total difficulties score ranges from 0 to 40 [26]. In this study, the extended version of SDQ was used. This version includes an impact assessment to evaluate how much and for how long the identified mental difficulties interfere with the child’s everyday life. A functional impairment score is calculated based on five items on whether the difficulties upsets or distresses the child and how much the difficulties interfere with home life, friendships, classroom learning, and leisure activities. Each item is scored on a scale from 0 to 2. To score 1 or 2, the interference from the difficulties in that domain must be assessed to either “quite a lot” or “a great deal” [23, 27]. Hence, the Impact score ranges from 0 to 10.

All parents responded to the SDQ in the beginning of the visitation process. A second response to the SDQ was obtained as a baseline measure for the children who at the end of visitation process were enrolled into the MMM trial. Youth aged ≥ 11 years old also answered the SDQ themselves. As the enrolled school-aged population was aged 5–16 years, the focus in this study was on parental-reported SDQ. Child reported SDQ results are available in Table S2 Supplemental Material.

In addition to the second SDQ measurement, a more thorough psychopathological assessment was performed for the youth who were enrolled in the MMM trial using The Developmental And Well-Being Assessment (DAWBA). DAWBA is an online questionnaire and rating techniques designed to generate Diagnostic and Statistical Manual of Mental Disorders (DSM-IV/5) psychiatric diagnosis in children and adolescents aged 5–17 years, covering the common emotional, behavioural and hyperactivity disorders [28, 29]. When scoring positive on screening questions, the interview opens for additional questions covering all the operationalized diagnostic criteria. Also, open-ended questions were administered to capture the respondent’s own description of the problems. All information was reviewed by experienced child and adolescent psychiatrists trained in DAWBA to decide on DSM-IV/5 diagnoses. When in doubt, consensus was reached among two or more raters. The Fleiss kappa coefficient for inter-rater reliability of the main groups of disorders was 0.65. The diagnoses were solely used to describe the psychopathology of the included youth for research purposes.

The Mood and Feelings Questionnaire (MFQ) [30] and Spence Children’s Anxiety Scale (SCAS) [31] were used in the visitation process to investigate the mental health problems. Both scales are specific psychopathological questionnaires focusing on depression and anxiety symptoms, respectively, unlike the SDQ, which has a general focus. In this study, we screened for different mental problems of the children, hence, we focused on the SDQ responses. The MFQ and SCAS responses are reported in Table S3 Supplemental Material together with Danish norms [31, 32].

### Age, sex, and socioeconomic factors

Information on age, sex, and socioeconomic factors were derived on an individual level from national registers. Socioeconomic factors included immigration status (being first- or second-generation immigrant), mother’s and father’s highest achieved education, number of children in the household, type of household, and household income. Individual level data of the background population were also used in this study. The background population was defined as all youth aged 6–16 years in the four municipalities on January 1, 2018. All register data used in this study were based on data from the year 2018.

### The visitation model

Referral to the visitation process was based on self-referral by help-seeking parents to ensure an easy and fast access to help. Information about the MMM trial and requirements for inclusion were published online in school intranets and municipality websites and given to local educational and health care professionals in the four municipalities including teachers, PPR psychologists, and general practitioners. No formal referral was required. The help-seeking parents were guided by professionals at the child’s school or in the municipality to contact the local PPR via telephone to sign up the child for assessment. An administrative assistant asked the parents to provide contact information (telephone and e-mail of child and both parents/guardians) and to return a form with written informed consent to take part in the web-based, digital data collection.

The visitation model was implemented during the period of the MMM trial in the four municipalities as part of standard care. The aim of the visitation was to identify the target population for an early treatment, as offered with the MMM intervention, during the trial period and thereafter. This was achieved through a systematic stratification of the youth with help-seeking parents. The youth were stratified into three groups: Stage 1, Stage 2, and Stage 3. The stratification process is referred to as the visitation model.

The visitation model consisted of two phases.

#### Phase 1: web-based initial assessments

First, the parents received a text message with link to answer the SDQ online. The SDQ responses and a screening cut-off algorithm were used to identify the youth deemed having mental health problems not severe enough to be offered an early treatment (Stage 1 group). The cut-off was based on age-matched populations in Denmark, Germany and the UK [27, 33, 34], so that scoring above the cut-off implied that the youth’s mental health state is ranked among the 10% of the most affected in these populations [35]. A score above the cut-off required SDQ specific scores of ≥ 1 in the SDQ Impact score combined with at least one of the following scores: total difficulties score of ≥ 14 and/or an Emotional problem score of ≥ 5 and/or a Behavioural problem score of at least 3. This cut-off was evaluated in a recent study as effective in identifying youth with mental health problems and a high risk of poor school outcomes years later [35]. The normal biopsychosocial development of children and adolescents in this age group include physical, social and emotional growth and changes, and the onset of puberty further accelerates the hormonal, emotional and bodily changes that may cause distress and temporary mental health problems. The minimum score on the impact score was included to ensure that the mental health problems were of such nature that they caused impairment for the youth. Youth ≥ 11 years old also answered the SDQ initially; these answers were, however, not used in the cut-off process.

If the parents’ responses to SDQ scored below the cut-off, they were automatically informed that the youth’s current mental health problems were not of sufficient severity that an intervention was required, and standard low-intensity offers like self-help and general counselling in the municipalities was considered. If the parents reported SDQ scores above the cut-off, they and their youth were automatically presented with MFQ and SCAS online. After having responded to SDQ, MFQ, and SCAS, the parents and the youth were invited to a meeting with a local PPR psychologist.

#### Phase 2: Meeting with the psychologist and psychopathological interview

The PPR psychologist reviewed the responses from the parents and youth to the SDQ, MFQ and SCAS before the meeting. At the meeting, the psychologist conducted a structured interview concerning the youth’s development, family and social situation, school attendance, learning problems, symptoms and functioning in daily life. The meeting also included a brief, semi-structured psychopathological interview with both youth and parent(s), inspired by the KSADS-PL [36] and ADIS-IV [37] but modified to screen for all the main groups of mental health disorders in childhood and adolescent. Based on the interview, an agreement was made between the psychologist, youth and parent(s) about the formulation of the youth’s “top problem” which they would like to address.

If the assessment revealed signs of severe developmental or severe mental disorder, including intellectual functional impairment, autism spectrum disorder (ASD), attention deficit/hyperactivity disorder (ADHD), psychotic disorder, eating disorder, obsessive–compulsive disorder, repeated self-harm, abuse or dependence of alcohol or psychoactive drugs, the youth was excluded from the trial and referred to CAMHS or other health care professionals for more comprehensive assessment and treatment (Stage 3 group). If the remaining youth did not meet any of the other exclusion criteria (see Table S1 Supplemental Material) including previous mental disorder diagnosis, informed consent of participations in the trial was obtained and the youth were included in the trial and a baseline assessment including SDQ was conducted (Stage 2 group).

### Data analysis

#### Stratification

This article focuses on the youth stratified to the three stage groups as defined earlier. The youth excluded due to other exclusion criteria (e.g., unable to participate in weekly session (see Table S1 Supplemental Material for full list of exclusions)) were not included in the analyses.

The parental responses to SDQ and the derived scores were used to characterize the help-seeking population and to analyse whether the visitation process succeeded in stratifying the youth into three categories with increasing severity of mental health problems. Danish norms from a population survey were used as reference values [34]. The youth-reported SDQ, as well as parental- and youth-reported MFQ and SCAS were analysed the same way and the results are available in Table S2 and S3 Supplemental Material.

The duration of the presented mental health problems was quantified as part of the SDQ with a question about the duration of the difficulties, where the parents could indicate if the difficulties have been present “less than a month”, “1–5 months”, “6–12 months”, or “Over a year”.

The youth that were included in the MMM trial (stage 2 group) were assessed with DAWBA. The diagnoses derived from this assessment are presented in the results section to explore the severity of mental health problems in relation to the diagnostic thresholds in this group of community treated youth.

#### Feasibility

To examine the feasibility of the visitation model, we analysed the duration of the visitation procedures and the mean changes in SDQ scores.

The time from the help-seeking parents responded to the questionnaires in the phase 1 online module until they had the phase 2 interview with the PPR psychologist analysed. The predetermined aim was a median time of 2 weeks for the process. This aim was decided based on a combination of being ambitious about providing fast assessment and being realistic about minimum of one parent being able to attend the interview during normal working hours.

For the youth included in the MMM trial, we analysed the differences in SDQ sub- and sum-scores between the beginning and end of the visitation process. This process was only possible for this specific group, as their parents answered SDQ again at the baseline of the trial after inclusion.

#### Representativeness

We compared characteristics between the three groups in visitation and the background population in the four municipalities to investigate possible inequity problems with the approach of the visitation model. The background population was defined as all youth aged 6–16 years old in the four municipalities on first of January 2018. Age, sex, and socioeconomic factors were compared. We expected a positive selection from the beginning of the visitation process towards youth from higher socioeconomic status groups, due to the need of parents to take the initiative of getting involved and answer questionnaires in the visitation process. Differences between the youth participating in the visitation and the background population are not necessarily due to the visitation model itself as it could also simply be due to the differences in prevalence of mental health problems among age-groups, sex, and socioeconomic groups. Thus, the findings will be interpreted based on the known variations in prevalence of mental health problems in two Danish cohorts [2, 35, 38]. If there was no inequity in access, we would expect to find that the population taking part in the visitation would have at least a similar proportion of youth with immigration background as the background population, that a larger proportion of the mothers in the visitation population would have a lower education, that a higher proportion of the youth in the visitation population would come from a household with a single parent, and that a lower proportion in the visitation population would come from a high income household. We expected to find a similar sex proportion in the visitation population as in the background population due to small sex differences in the rate of mental health problems, while we expected to observe a higher proportion of older youth in the visitation population due to increasing incidence rates of mental health problems with age.

#### Statistical analysis

Pearson’s Chi squared test and Student’s *t* tests were used to compare groups for categorical and continuous variables, respectively. Shapiro-Francia test was used to test for normality. The sub-scores of the SDQ (not Total difficulties and Impact score) failed the normally test. As a robustness test, Mann–Whitney *U* test was utilized in the comparison of these scores. Similarly, when paired *t* test was used, Wilcoxon matched-pairs signed-ranks test was used for robustness check. This was not possible when comparing with Danish norms, as these data were only available in aggregated numbers. All robustness checks did, however, find the results of the Student’s *t* tests robust. *P*-levels < 0.05 are referred to as statistically significant throughout. To ensure data confidentiality, Statistics Denmark does not allow output for cells containing data from < 4 individuals, and we therefore do not report exact results in such cases, but merely report that the number of individuals is < 4. All analyses were performed in STATA-15 [39].

## Results

### The visitation model

**Fig. 1:**
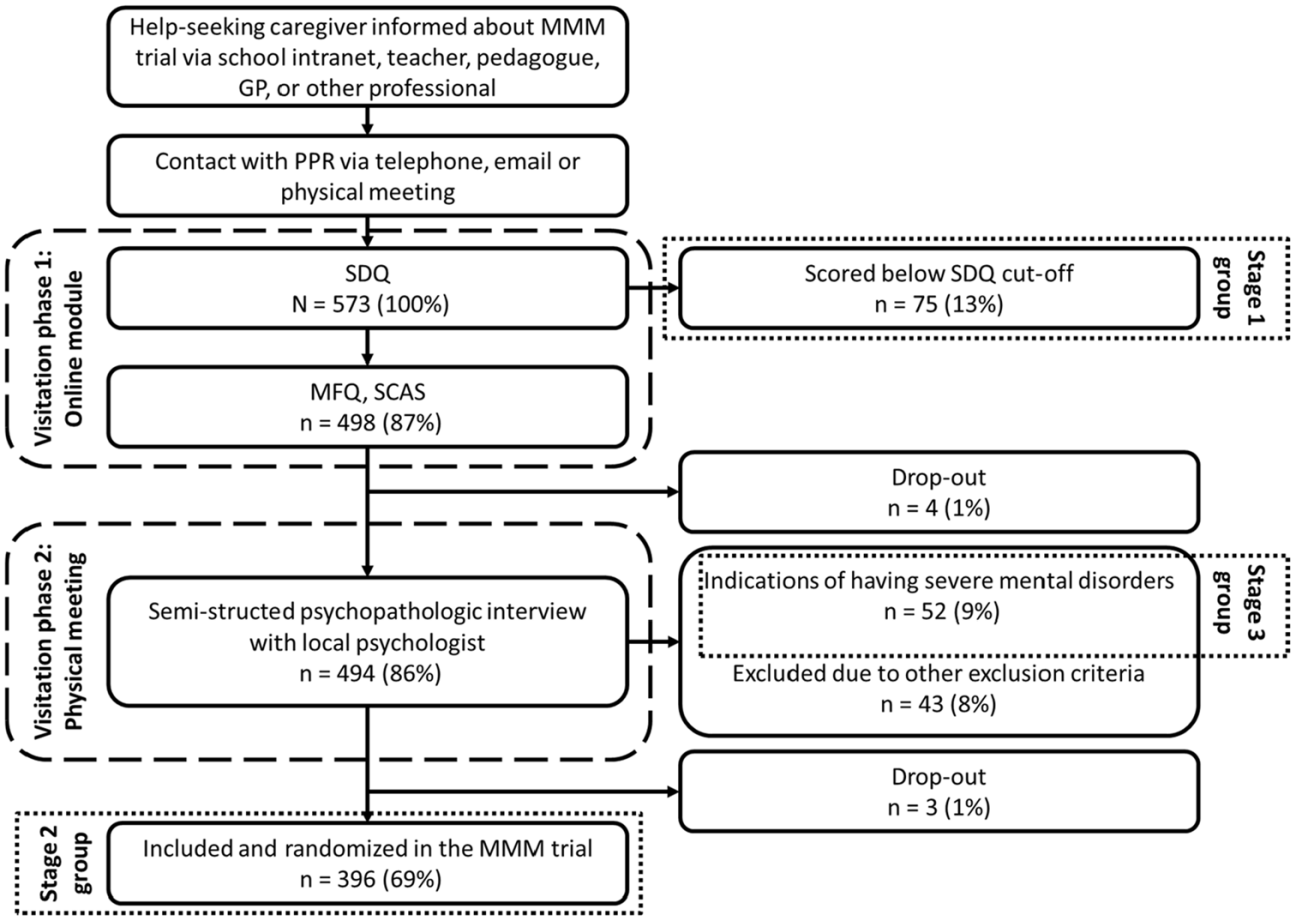
Flowchart of visitation process. *PPR* Educational and psychological counselling. *SDQ* The Strength and Difficulties Questionnaire, *MFQ* The Mood and Feelings Questionnaire, *SCAS* Spence Children’s Anxiety Scale, *MMM* Mind My Mind

Altogether, 573 youth/their parents contacted their local PPR, gave consent and contact information, and subsequently entered the online module of phase 1 in the visitation process and responded to the SDQ. The flow of the visitation process and the delineation of the stratification groups are illustrated in Fig. 1. Seventy-five (13%) of the youth scored below the cut-off. These youth constitute the stage 1 group in the stratification. In phase 2 of the visitation process, 52 (9%) youth were excluded due to indications of severe mental disorders, and these youth constitute the stage 3 group. Furthermore, 43 youth were excluded based on other exclusion criteria. Seven children dropped out for unknown reasons. Finally, 396 (69%) youth were included in the MMM trial, and these youth constitute the stage 2 group in the stratification.

The youth in Stage group 1, 2, and 3 had a mean age of 10.2 years (median 10.0) years, and 47% were girls. For sex, age, immigration status, household type, and household income, we found no statistically significant differences between the three groups (Table 1). For the mother’s highest achieved education, we found a statistically significant difference between group 2 and 3. A higher proportion of youth in group 3 had mothers with a lower secondary education. Despite not being statistically significantly different, the same tendency was found for group 1 versus 3. For the father’s highest achieved education, we found a statistically significant difference between group 1 and 3. This is due to a large proportion of fathers in group 1 having an upper secondary education. The findings do not seem to indicate any systematic bias in the selection of the groups, as they were not consistent between variables.

**Table 1:** Socio economic characteristics of the three staging groups

	Stage 1 group *n* = 75 (74^a^)	Stage 2 group *n* = 396	Stage 3 group *n* = 52
Sex, female, *n* (%)	34 (45)	190 (48)	21 (40)
Age, years, mean (SD)	10.1 (0.30)	10.2 (0.12)	10.2 (0.40)
1st or 2nd generation immigrant, *n* (%)	NA	5 (1)	NA
Mother’s highest education, *n* (%)		^+^	
Lower secondary	5 (7)	27 (7)	10 (19)
Upper secondary	30 (41)	152 (41)	18 (35)
Short cycle tertiary, Bachelor or equivalent	33 (45)	186 (48)	20 (38)
Master or equivalent	6 (8)	25 (6)	4 (8)
Father’s highest education, *n* (%)	^*^		
Lower secondary	8 (13)	53 (15)	9 (19)
Upper secondary	41 (64)	178 (49)	19 (40)
Short cycle tertiary, Bachelor or equivalent	10 (15)	91 (25)	18 (38)
Master or equivalent	5 (8)	38 (11)	NA
No. of children in household, *n* (%)	^$^		
1	24 (32)	67 (17)	6 (12)
2	30 (41)	235 (59)	30 (58)
3 +	20 (27)	94 (24)	16 (31)
Household type, *n* (%)			
Single woman	15 (20)	90 (23)	15 (29)
Couple	47 (64)	276 (70)	32 (62)
Other constellations (single male or more than one family)	12 (16)	30 (8)	5 (10)
Household income before tax, *n* (%)			
0–500,000 DKK	24 (32)	112 (28)	21 (40)
> 500,000–1,000,000 DKK	40 (54)	235 (59)	23 (44)
> 1,000,000 DKK	10 (14)	49 (12)	8 (15)

^a^One child is not identifiable in the national register, hence only sex and age were available. *NA* Not applicable due to the combination of low number and data confidentiality

*Group 1 is statistically significant different from group 3 on a *P*-level < 0.05

^+^Group 2 is statistically significant different from group 3 on a *P*-level < 0.05

^$^Group 1 is statistically significant different from group 2 and 3 on a *P*-level < 0.05

### The stratification

When we analysed the parents’ responses to the SDQ, we found that all three groups had a statistically significant worse mean Total difficulties score and Impact score compared to the Danish norms. For the sub-scores, this was also the case for Emotional problems, Behavioural problems, and Hyperactivity, while only group 2 and 3 had statistically significant worse scores than the Danish norm in the sub-scores Peer problems and Pro-social behaviour. All mean scores, except Emotional problems, followed the same pattern of worse mean scores from group 1 to group 2, and from group 2 to group 3. For Emotional problems, group 2 had a non-significant higher mean score compared to group 3. Group 1 had statistically significant better mean scores in all scoring categories compared to group 2 and 3. Compared to group 3, group 2 had significant better mean scores in Hyperactivity, Peer-problems, Total difficulties and Impact score (Table 2). The mean difference in impact score from group 1 to the group 2 was 3.1 (95% CI: 2.7–3.6), and 1.0 (95% CI: 0.5–1.6) from group 2 to group 3.

**Table 2:** Psychopathology of the youth at visitation

	Stage 1 group *n* = 75	Stage 2 group *n* = 396	Stage 3 group *n* = 52	Danish norms^1^ *N* = 3146
Psychopathology				
Strength and difficulties questionnaire, mean (SD)				
Emotional problems	3.5 (2.0)^*^	7.0 (2.4)	6.4 (2.4)	2.3 (2.3)^$^
Behavioural problems	1.4 (1.3)^*^	2.8 (2.0)	3.3 (1.8)	0.9 (1.3)^$^
Hyperactivity	3.1 (2.1)^*^	5.0 (2.8)^+^	6.3 (2.3)	2.5 (2.5)^$^
Peer problems	1.6 (1.6)^*^	2.8 (2.1)^+^	3.7 (2.6)	1.3 (1.7)^£^
Pro-social behaviour	8.4 (1.9)^*^	7.6 (2.1)	7.0 (2.3)	8.7 (1.5)^£^
Total difficulties score	9.6 (4.1)^*^	17.5 (5.1)^+^	19.7 (5.1)	7.1 (5.8) ^$^
Impact score	1.1 (1.6)^*^	4.2 (1.9)^+^	5.3 (2.2)	0.6 (1.6) ^$^

^1^Danish norms of children aged 6–17 from Arnfred et al.[34]

^*^Group 1 is statistically significantly different from group 2 and 3 on a *P*-level < 0.05 tested with both *t* test and Mann–Whitney *U* test

^+^Group 2 is statistically significantly different from group 3 on a *P*-level < 0.05 tested with both *t* test and Mann–Whitney *U* test

^$^Danish norms are statistically significantly different from group 1, 2, and 3 on a *P*-level < 0.05 tested with *t* test

^£^Danish norms are statistically significantly different from group 2 and 3 on a *P*-level < 0.05 tested with *t* test

For the majority of the youth (82%), the mental health problems had lasted more than a year, while only 39 youth (7%) had experienced problems for less than 5 months. No statistically significant differences were found between the three groups, but there was a tendency to a shorter duration of problems in group 1.

At the DAWBA assessment of the 396 youth who were included in the MMM trial, 317 (80%) fulfilled the criteria of at least one DSM-IV/5 diagnosis and 26% fulfilled diagnostic criteria from more than one diagnostic group. More than half of the 396 youth (56%) suffered from one or more anxiety disorder, 97 (24%) suffered from a behavioural disorder, and 58 (15%) from a depressive disorder. The group of youth with neurodevelopmental disorders comprised individuals who fulfilled the criteria for ADHD (*n* = 46), ASD (*n* = 3), and/or tics disorders (*n* = 11). In total, 57 (14%) of the 396 youth had at least one of these neurodevelopmental disorders (Table 3).

**Table 3:** Psychopathology of the youth included in the mind my mind

Strength and difficulties questionnaire, mean (SD)			
	Visitation score	Baseline score	Paired *t* test between visitation and baseline score, *P* value^1^
Emotional problems	7.0 (2.4)	6.4 (2.5)	< 0.001
Behavioural problems	2.8 (2.0)	2.6 (2.0)	< 0.001
Hyperactivity	5.0 (2.8)	4.7 (3.0)	0.049
Peer problems	2.8 (2.1)	2.5 (2.1)	< 0.001
Pro-social behaviour	7.6 (2.1)	7.6 (2.0)	0.566
Total difficulties score	17.5 (5.1)	16.3 (5.5)	< 0.001
Impact score	4.2 (1.9)	4.2 (2.4)	0.550
Days between parents responding to questionnaire at visitation and baseline, mean (SD)			30.9 (20.7)
DSM-IV/5 Mental disorders based on the development and well being assessment (DAWBA), *n* (%)			
Anxiety disorder	220 (56)		
Depressive disorder	58 (15)		
Behaviour disorder	97 (24)		
Neurodevelopmental disorder	57 (14)		
Any disorder	317 (80)		
Comorbidity, ≥ 2 disorders	102 (26)		

*N* = 396. ^1^As a robustness test Wilcoxon matched-pairs signed-ranks tests were performed for all subscales which resulted in similar statistical significance levels

### Feasibility

For the 494 children who continued to phase 2 of the visitation process, the mean time in visitation from when the online module of phase 1 was finalized to the interview with the PPR psychologist constituting phase 2 was 18.7 (SD: 19.7) days. The median time was 15 days, and 95% had the interview within 36 days.

The 396 youth who were included in the MMM trial had a mean time between the two SDQ assessments of 30.9 days (SD: 20.7) (Table 3). For all, but Prosocial and Impact score, mean scores were significantly improved at the baseline responses compared to the time of visitation. The mean Impact score did not change between the two points in time, but the variance in the score increased. At baseline, 8% had the minimum Impact score of 0 and 5% had a score of 9 or 10, compared to 0% and 3%, respectively, at the time of visitation.

### Representativeness

Comparing the distribution of sex, age, and socioeconomic variables between the total population of the three groups in the visitation to the background population in the four municipalities, we found statistically significant differences for all variables except for sex and father’s education level. For household number of children, type, and income, we found minor differences, and in the direction expected from the cohort studies [35, 38]. Age was significantly lower in the visitation population relative to the background population, due to a lower proportion of adolescents aged 14–16 years in the visitation population. This group represents 10% compared to 29% in the background population. The proportion of 1st and 2nd generation immigrants across the two populations differed significantly with few in the visitation population having an immigrant background. Moreover, the background population had a higher proportion of mothers with lower secondary as the highest education level (Table 4).

**Table 4:** Socioeconomic characteristics of the visitation population and the background population

	Total of group 1–3 *n* = 523 (522^a^)	Background population^1^ *n* = 32,814
Sex, female *n* (%)	245 (47)	15,989 (49)
Age, mean (SD)	10.2 (0.11)	11.2 (3.12)^*^
1st or 2nd generation immigrant, *n* (%)	NA	3,198 (10)^*^
Mother’s highest education, *n* (%)		^*^
Lower secondary	42 (8)	4,900 (15)
Upper secondary	200 (39)	12,368 (39)
Short cycle tertiary, bachelor or equivalent	239 (46)	11,678 (37)
Master or equivalent	35(7)	2,939 (9)
Father’s highest education, *n* (%)		
Lower secondary	70 (15)	5,953 (19)
Upper secondary	238 (50)	15,022 (48)
Short cycle tertiary, Bachelor or equivalent	119 (25)	7,015 (23)
Master or equivalent	NA	3,029 (10)
Children in household, *n* (%)		^*^
1	97 (19)	5,152 (16)
2	295 (57)	16,030 (49)
3 +	130 (25)	11,632 (35)
Household type based on adults, *n* (%)		^*^
Single woman	120 (23)	5637 (17)
Couple	355 (68)	22,964 (70)
Other constellations	47 (9)	4213 (13)
Household income before tax, *n* (%)		^*^
0–500,000 DKK	157 (30)	9379 (29)
> 500,000–1,000,000 DKK	298 (57)	16,186 (49)
> 1,000,000 DKK	67 (13)	7249 (22)

^a^One child is not identifiable in the national register, hence only sex and age were available. *NA* not applicable due to the combination of low number and data confidentiality

*The total population in the visitation is statistically significant different from the background population on a *P*-level < 0.05

^1^All children aged 6–16 in the four participating municipalities

## Discussion

Using a stage-based stepped-care approach the visitation model succeeded in defining three groups of youth with increasing severity of mental health problems based on parental-reported SDQ. The MFQ and SCAS showed the same tendencies in the results using both parent- and self-reported scores. The anxiety specific scores (SDQ emotional problems and SCAS scores) were not statistically different in group 2 and 3. This was, however, acceptable, as the MMM intervention was designed to handle high levels of anxiety if there is no profound comorbidity. We found that all three groups were more affected by mental health problems than the average Danish child. The majority of the youth who took part in the visitation were heavily affected by their mental health problems, with a total of 10.5% assessed as having signs of severe mental health disorders, and with further 61% of the youth fulfilling the criteria of a mental disorder diagnosis. These findings falls in line with findings of previous studies, in which young people seeking help in community-based services often have more severe mental health problems than first-line services are designed to address [40]. The present study highlights the urgent need for systematic identification of mental health issues in youth, so that interventions can be delivered to help this group of youth, who do not receive adequate or timely care today. Findings by Copeland et al. [6] and Wolf et al. [35] underline that without an intervention, these youth are likely to suffer from long-term adverse consequences.

The investigation of potential inequity in access with the visitation model highlighted three major concerns. Comparing the youth in the visitation model with the background population from the four municipalities, we found indications of a selection that disfavoured youth with immigration background as well as children of mothers with lower level of education. The difference in proportion of youth with immigration background cannot be explained by the exclusion criteria of no parent speaking Danish as only one child was excluded due to this (see Table S1 Supplemental Material), or by the prevalence, as 1st and 2nd generation immigrants have at least the same prevalence of mental health problems [35]. The visitation model seemed to be biased towards youth having parents with more resources, which could be due to the self-referral approach. Another issue identified in the comparison between the population that took part in the visitation and the background population was that the proportion of adolescents aged 14–16 years who took part in the visitation was low compared to the background population despite our expectation of a higher proportion due to increasing incidence rates of mental health problems during adolescence [38]. This finding points towards a limitation of the visitation model in recruiting youth with mental health problems in their early teenage years. This limitation could be due to two factors. First, internalizing mental health problems are more common in the older age group [38] and this type of problems might not be noticed by parents and/or teachers as often as other types of problems, especially externalizing ones. Furthermore, the visitation model was advertised with the MMM intervention. This material could perhaps have had a form that appealed more to parents and younger children, making the older age group less likely to take action themselves and professionals less likely to inform this group. Other models aiming specifically at adolescents typically take efforts to make themselves directly accessible for this age group [40]. In future implementation of the visitation model, careful information, nudging and other helpful initiatives to enrol the youth of mothers with lower education, youth with immigration background, and youth in the age group 14–16 years into the visitation should be considered to avoid inequity in mental health care and to optimize the visitation model. The approach with open self-referral by help-seeking parents should, however, still be maintained as it clearly succeeded based on the findings of all three groups being more affected by mental health problems than the average Danish youth, and the majority were in need of an early intervention or further psychiatric assessment.

The presented visitation model had an acceptable duration from the online module to the meeting and assessment with the local psychologist. The median of 15 days does, however, show that there is room for improvement as half the youth experienced longer time than the pre-stated aim of two weeks. However, considering that more than nine out of ten youth had experienced their mental health problems for more than 5 months, the duration of the visitation process seems acceptable. The SDQ score change from visitation to baseline showed that the youth on average seemed to improve slightly during the visitation process.

Another important issue is the latency period from identification and assessment of problems to access to treatment [5]. An early systematic assessment and staging of the youth, as proposed with this model, can contribute to shorten wait times by ensuring that the correct target group reaches the different interventions in a timely way. With no systematic approach previously in place, it is likely, however, that more youth in need of an intervention will be identified initially, as many youth in need currently are not being identified and offered early treatment [35]. This means that the full effect of implementing the visitation model may likely only be achieved over time.

A limitation of the presented visitation model is that it only focuses on a segment of the full framework of mental health care [16]. Important areas, such as mental health literacy and universal prevention, are not addressed. Also, the actions and procedures in CAMHS are beyond the scope of this visitation model. An important aspect that was not addressed here is the collaboration across mental health care services within and beyond the health care system. A fast handover of youth with signs and symptoms of severe mental disorders is crucial to assure effectiveness of the early identification and visitation model. Also, standard procedures for the youth who score below the SDQ cut-off should be further formalized to ensure that all help-seeking parents receive adequate support. This should include information on how to act if the mental health problem intensifies. Another limitation of the visitation model is that it does not directly address all types of mental health problems. If youth with these problems are identified in the face-to-face interview in the visitation process, the involved psychologist can, however, ensure referral to the appropriate professionals. A methodological limitation was that the diagnostic assessment with DAWBA was only carried out for stage 2 group, and not the others.

A strength of the visitation model is that its focus is not limited to a single type of disorder. The model allows for the link with for example, the transdiagnostic MMM intervention that focuses on the most prevalent childhood mental health problems: anxiety, depression, and behavioural problems. This feature makes the visitation model more feasible and sustainable for wide-scale implementation. Even in smaller geographical areas, there will often be a sufficient number of youth suffering from these kind of mental health problems to render the systematic approach effective. Compared to having identification and stratification models for each specific area, the transdiagnostic approach is also likely to incur lower implementation and running costs.

In summary, this study provides evidence that the visitation model based on a stage-based stepped-care approach with systematic identification and stratification of help-seeking school-aged children and adolescents with mental health problems is possible to carry out successfully in a community setting. It appears realistic to reduce the observed inequity in access by strengthening the outreach to youth with immigration background, youth of mothers with lower level of education and youth aged 14–16 years before full, wide-scale implementation.

## Supplementary Information

Below is the link to the electronic supplementary material.

Supplementary file1 (DOCX 27 KB)

## Data Availability

The data utilized in the current study are defined as sensitive personal data, and cannot be shared publicly due to existing data protection laws in Denmark, and imposed by the Danish Data Protection Agency. The collected data are uploaded and linked to data from the Danish National registers and analysed through Statistics Denmark (https://www.dst.dk/en).
