# Supplementary material for: Systematic identification and stratification of help-seeking school-aged youth with mental health problems: a novel approach to stage-based stepped-care
Source: Eur Child Adolesc Psychiatry. 2021 Jan 18;31(5):781–93. doi: 10.1007/s00787-021-01718-5 (PMC9142415; doi:10.1007/s00787-021-01718-5)
Supplement: Supplementary file 1 — Supplementary file1 (DOCX 27 KB) [file 787_2021_1718_MOESM1_ESM.docx]

**Supplemental Material**

**S1: Exclusion criteria in phase 2**

|  | **N (%)** |
| --- | --- |
| Not aged 6–16 years and in 0-9th grade | (2) |
| Indications that the child may have a severe mental disorder | 52 (55) |
| Top problem not in the domains of anxiety, depressive symptoms or behavioral problems | 51 (54) |
| No parents understand and speak Danish sufficiently to participate in the treatment | 1 (1) |
| Indications of intellectual functional impairment, severe learning difficulties or other special needs | 14 (15) |
| A prior diagnosis of any developmental or mental disorder | 8 (8) |
| The child and/or parents are unable to participate in weekly sessions | 8 (8) |
| Parents does not want their child to participate | 30 (32) |
| **Total** | **95 (100)** |

Note: One child can meet more than one exclusion criteria.

**S2: The Strength and Difficulties Questionnaire (SDQ) self-reported**

|  | Group 1  Below SDQ algorithm cut-off  n = 9 | Group 2  Included and randomized  n = 178 | Group 3  Indications of having severe mental disorders  n = 20 | Danish norms^1^  N = 1583-1609 |
| --- | --- | --- | --- | --- |
| **Psychopathology** | | | | |
| **The Strength and Difficulties Questionnaire, mean (SD)** | | | |  |
| Emotional problems | 6.6 (2.9) | 5.9 (2.6) | 5.3 (2.7) | 2.7 (2.3)^$^ |
| Behavioural problems | 1.8 (1.2) | 2.4 (1.7) | 2.9 (1.6) | 1.4 (1.5)^£^ |
| Hyperactivity | 5.0 (3.3) | 4.4 (2.7)^+^ | 5.8 (2.9) | 3.5 (2.3)^£^ |
| Peer problems | 2.7 (1.7) | 2.7 (2.0) | 3.4 (2.0) | 1.8 (1.7)^£^ |
| Pro-social behaviour | 7.8 (1.9) | 7.7 (1.5) | 7.3 (2.0) | 7.6 (1.7) |
| Total difficulties score | 16.0 (5.9) | 15.3 (5.5) | 17.3 (6.1) | 9.4 (5.5)^$^ |
| Impact score | 1.6 (1.6) | 2.4 (2.3) | 2.7 (2.0) | 0.4 (1.2)^$^ |

Note: ^1^Danish norms of children aged 6-17 from Arnfred et al.[34]. *Group 1 is statistically significant different from group 2 and 3 on a p-level <0.05 tested with both t-test and Mann-Whitney U test. ^+^Group 2 is statistically significant different from group 3 on a p-level <0.05 tested with both t-test and Mann-Whitney U test. ^$^Danish norms are statistically significant different from group 1, 2, and 3 on a p-level <0.05 tested with t-test. ^£^Danish norms are statistically significant different from group 2 and 3 on a p-level <0.05 tested with t-test.

**S3: The Mood and Feelings Questionnaire (MFQ) and Spence Children’s Anxiety Scale (SCAS) at visitation**

|  | Group 2  Included and randomized | Group 3  Indications of having severe mental disorders | Danish norms^1^ |
| --- | --- | --- | --- |
| **MFQ^2^** | | | |
| Parent reported, mean (SD) | 21.9 (11.5)^*^ | 27.5 (13.2) | 7.1 (7.8)^$^ |
| N | 395 | 52 | 703 |
| Self-reported, mean (SD) | 21.8 (12.2)^*^ | 29.7 (15.1) | 13.2 (10.8)^$^ |
| N | 340 | 40 | 992 |
| **SCAS^3^** | | | |
| Parent reported, mean (SD) | 35.6 (16.5) | 33.1 (17.9) | 14.4 (10.3)^$^ |
| N | 396 | 51 | 537 |
| Self-reported, mean (SD) | 38.2 (17.6) | 41.4 (22.2) | 22.0 (14.3)^$^ |
| N | 339 | 40 | 972 |

Note: ^1^Danish norms for MFQ is of children aged 9-17 from Eg et al.[32]. Danish norms for SCAS is of children aged 7-17 from Arendt et al. [31]. ^2^MFQ parent reported has 34 items and a score range from 0 to 68. MFQ self-reported has 33 items and a score range from 0 to 66 [32]. ^3^SCAS parent reported has 39 items and a score range from 0 to 117. SCAS self-reported has 44 items and a score range from 0 to 132 [31]. *Group 2 is statistically significant different from group 3 on a p-level <0.05 tested with both t-test and Mann-Whitney U test. ^$^Danish norms are statistically significant different from group 2, and 3 on a p-level <0.05 tested with t-test.
